# Valve surgery in combination with cryoablation in the treatment of atrial fibrillation

**DOI:** 10.12669/pjms.346.15537

**Published:** 2018

**Authors:** Weiwei Cai, Jie Hu, Hua Wang, Song Chen, Guijun Zhu, Xingpeng Chen

**Affiliations:** 1 *Weiwei Cai, Luoyang Central Hospital Affiliated to Zhengzhou University, Luoyang, Henan, China*; 2 *Jie Hu, Luoyang Central Hospital Affiliated to Zhengzhou University, Luoyang, Henan, China*; 3 *Hua Wang, Luoyang Central Hospital Affiliated to Zhengzhou University, Luoyang, Henan, China*; 4 *Song Chen, Luoyang Central Hospital Affiliated to Zhengzhou University, Luoyang, Henan, China*; 5 *Guijun Zhu, Luoyang Central Hospital Affiliated to Zhengzhou University, Luoyang, Henan, China*; 6 *Xingpeng Chen, Luoyang Central Hospital Affiliated to Zhengzhou University, Luoyang, Henan, China*

**Keywords:** Heart valve disease, Cryoablation, Atrial fibrillation

## Abstract

**Objective:**

To investigate the effectiveness and safety of valve surgery in combination with cryoablation in the treatment of atrial fibrillation.

**Methods:**

Fifty patients who needed to undergo valve surgery because of heart valve disease and sixty-five patients who needed to undergo valve surgery in combination with cryoablation were selected as the research subjects. The perioperative technical characteristics, postoperative curative effect and complications were compared between the two different treatment modalities in our hospital between October 2013 and November 2015.

**Results:**

No significant differences were observed between the two groups in valve surgery mode, valve type and postoperative length of hospital stay (P>0.05), but the duration of extracorporeal bypass and aorta occlusion of the observation group was longer than that of the control group (P<0.05). The 3-month, 6-month and one-year atrial fibrillation cardioversion rates of patients in the observation group were superior to those of the control group, and the differences were notable (P<0.05). The improvement of left atrial diameter of the observation group was also superior to that of the control group (P<0.05). The incidence of postoperative complications of the two groups had no significant difference (P>0.05).

**Conclusion:**

Valve surgery in combination with cryoablation has favorable short-term clinical effect in the treatment of heart valve disease in combination with atrial fibrillation, which is worth promotion.

## INTRODUCTION

Heart valve disease in combination with atrial fibrillation is a common frequently-occurring disease with high disability rate and fatality rate.^1^ Atrial fibrillation is the most common arrhythmia associated complication of patients with valvular heart disease; patients with mitral valve disease especially have the highest incidence of atrial fibrillation, about 30%.^2^ It cannot only affect the cardiac function, but also increase risks of cerebral stroke and the perioperative death rate.^3^,^4^ An investigation of Chugh et al. suggested that 596.2 among every 100 thousand of males and 373.1 among every 100 thousand of females across the world in 2010 suffered from it which suggested a remarkable increase compared to 1990.^5^ Previous treatment methods for atrial fibrillation include administration of antiarrhythmic drugs, electrical conversion, radiofrequency catheter ablation and surgery,^6^,^7^ among which surgery has been an effective treatment method. Cox-Maze III surgery, a traditional atrial fibrillation therapy, has disadvantages of high operation difficulty, severe trauma and long operation duration, though it can significantly improve sinus rhythm cardioversion rate. Those defects can produce adverse effect on the recovery of body function after surgery and limit the application and promotion of Cox-Maze III surgery in clinics; therefore it has been gradually replaced by energy treatment.^8^,^9^ Currently radiofrequency ablation and cryoablation are the most frequently used energy therapies. Later the potential safety hazard of radiofrequency ablation was discovered, Cox JL et al. pointed out the high feasibility and low invasiveness of cryoablation as a new energy therapy for atrial fibrillation and explained the key points and considerable success rate of the treatment.^10^

In the present study, the safety and effectiveness of valve surgery in combination with biatrial cryoablation in the treatment of atrial fibrillation was studied with the intention to offer a reference for the promotion of valve surgery in combination with biatrial cryoablation.

## METHODS

### General Materials

Patients who were diagnosed as valvular heart disease in combination with atrial fibrillation in our hospital between October 2013 and November 2015 were retrospectively analyzed. Fifty patients who underwent valvular surgery only were selected and set as a control group, while sixty-five patients who underwent valvular surgery and biatrial cryoablation in the same period were selected as an observation group. It was their first time to undergo cardiac surgery. The baseline data and relevant clinical characteristics of patients in the two groups were collected and compared. The control group included 26 males and 24 females, and their average age was (56.2±6.1) years; atrial fibrillation has lasted (5.6±2.1) years, which was determined as long-standing persistent; the diameter of the left atrium was (57.1±6.9) mm, and the left ventricular ejection fraction (LVEF) was (59.8%±4.9%). As regards the cause of bicuspid valve in the control group, 33 cases were induced by rheumatic heart disease, and 17 cases were induced by degenerative change. There were 20 cases of mitral stenosis, 17 cases of mitral valve insufficiency, 13 cases of mitral stenosis in combination with mitral valve insufficiency, and 19 cases of mild and medium tricuspid incompetence. The observation group included 36 males and 29 females; and their average age was (55.8±6.4) years; atrial fibrillation has lasted (5.5±2.2) year), which was determined as long-standing persistent; the diameter of the left atrium was (57.4±7.0) mm, and the left ventricular ejection fraction (LVEF) was (60.4%±5.0%). As regads the cause of bicuspid valve in the control group, 45 cases were induced by rheumatic heart disease, and 20 cases were induced by degenerative change. There were 27 cases of mitral stenosis, 21 cases of mitral valve insufficiency, 16 cases of mitral stenosis in combination with mitral valve insufficiency, and 24 cases of mild and medium tricuspid incompetence. As regards general data between the two groups it had no statistical significance (P>0.05); hence the results were comparable. All procedures were done with the approval of the ethics committee of our hospital, and taking informed consent from patients.

### Inclusive and Exclusive Criteria

Patients who had valvular heart disease in combination with atrial fibrillation, aged between 18 and 70 years, had inner diameter of long-axial section of the left atrium not larger than 70 mm, and had LVEF not smaller than 45% were enrolled. Those who had atrial ectopia, abnormal atrioventricular connection and ventricular main artery connection, severe coronary artery lesions which required coronary artery bypass grafting, or congenital heart disease such as septal defect, patent ductus arteriosus and anomalous pulmonary venous drainage, who were unable to cooperate to fulfill various follow-up requirements, who were attending other clinical trials, who had severe respiratory insufficiency and insufficiency of important organs such as kidney and liver, who were considered as not suitable for participating in the study or who had undergone re-thoracotomy were excluded.

After superior and inferior vena cava bypass and ascending aorta block were completed, an incision was cut from suprasternal fossa to the 4th intercostal space, and then the sternum was transversed. Upon commencing extracorporeal circulation, a longitudinal left atriotomy was made through the right atrium-atrial septum. For patients who needed aortic valve replacement, an aortic root incision was made. The left aurcle was processed by extracardiac ligation using double No.10 silk thread and intracardiac suture using 4-O Prolene thread. Minimally invasive mitral valve surgery under thoracoscope was initiated with a 6-cm incision at the right anterolateral 4th intercostal space, followed by establishing femoral artery and vein bypass and filling surgery field with carbon dioxide. Mitral valve surgery was performed through a left atrial incision; the left aurcle was closed by intracardiac suture using 4-0 Prolene thread. Tricuspid valvuloplasty was performed through right atrial incision.

Patients in the observation group underwent biatrial cryoablation in addition to the same treatment as the control group. The treatment procedures were as follows. A mental probe (Atricure Inc., USA) was rapidly cooled to - 60°C under the action of a refrigerating machine and dinitrogen tetroxide and completely in contact with endocardial tissue to produce damage line which could effectively block electric excitement conduction. Patients in the observation group underwent first left atrial cryoablation and then right atrial cryoablation under aortic occlusion; followed by valve surgery.

**Fig.1:**
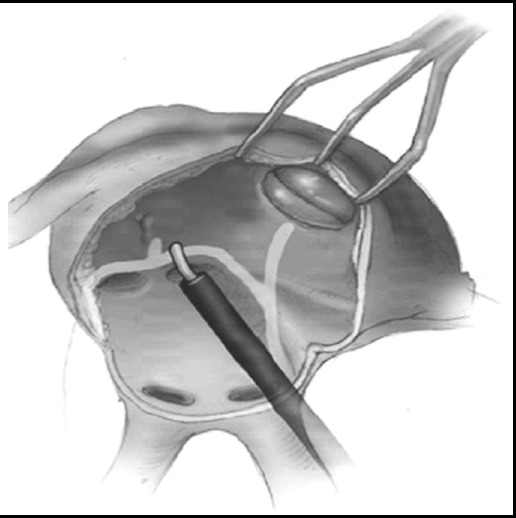
The left atrial cryoablation line.

Left atrial cryoablation was performed from the opening of the right upper pulmonary vein to the midpoint of the left upper pulmonary vein opening via left atrial top, from the opening of the right lower pulmonary vein to the midpoint of left pulmonary vein opening, from the opening of the right lower pulmonary vein to the left auricle and from the right pulmonary vein opening to mitral annulus. Every line was cryoablated for 120s. The sketch map is shown in Fig.1.

**Fig.2:**
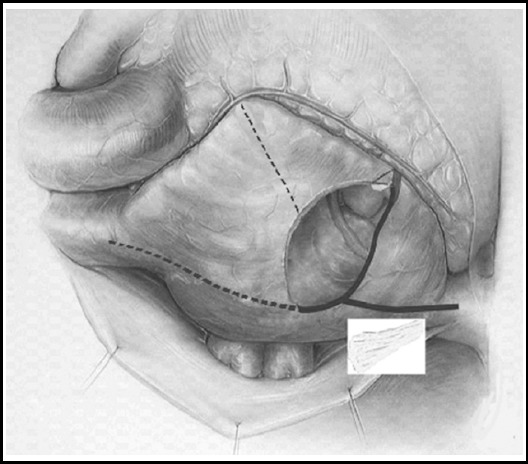
The right atrial cryoablation line.

The sketch map of right atrial cryoablation is shown in Fig.2. Right atrial lateral longitudinal incision was cut from the midpoint of interatrial groove towards tricuspid annulus. A cryoablation line was made at the junction of the front end of the incision and tricuspid annulus. The second cryoablation line was made at the junction of the midpoint of interatrial groove and tricuspid annulus via atrial septum and lower edge of coronary sinus ostium. Then cryoablation lines were made at the rear walls of superior vena cava and inferior vena cava respectively. After right auricle was removed, a cryoablation line was made at the junction of the incisal edge of right auricle and tricuspid annulus.

All the patients were implanted temporary pacing leads. Temporary pacemaker was used when heart rate was lower than 70 times/min; the pacing frequency was set as 90 times/minute. Pacing stopped if rhythm of the heart recovered. When the patients were able to eat food, 200 mg of cordarone (approval number: H19993254; Hangzhou Sanofi Pharmaceutical Co., Ltd., China) was orally administrated, once each day, and 2.5 mg of warfarin (approval number: H31022123; Shanghai Sine Pharmaceutical Laboratories Co., Ltd., China); the dose could be adjusted according to PT and International Normalized Ratio (INR). After discharge, the patients continued to take cordarone, 100~200 mg daily, once daily for three to six months. The cardiac rhythm was monitored using an electrocardiogram monitor in the first three days after surgery. 12-lead electrocardiogram was recorded before discharge, i.e. in the first seven days after surgery. After discharge, the cardiac rhythm of the patients was observed by reviewing 12-lead electrocardiogram in the 3^rd^ month, 6^th^ month and 12^th^ month after surgery. Atrial fibrillation was considered as recurrent if the duration of atrial fibrillation lasted for 30 seconds during the follow up. For patients who underwent mitral valvuloplasty or bioprosthetic valve replacement in the same period, warfarin was orally taken for six months to maintain INR at 2.0 if sinus rhythm restored; for patients who underwent mechanical valve replacement, INR was maintained between 1.7 and 2.5 regardless of the existence of atrial fibrillation.

### Statistical Analysis

Data were statistically processed using SPSS ver. 20.0. Measurement data were verified for normal distribution using Kolmogorov-Smirnov method; if the data was distributed normally, then they were expressed as mean ± standard deviation; if the data was distributed normally, then it was expressed in forms of median and inter-quartile range. Dichotomous data and ranked data were expressed by percentage. Comparison of measurement data between groups was performed using two-sample t test. Comparison of dichotomous data between groups was performed using Chi-square test. Comparison of ranked data between groups was carried out using Willcoxon rank sum test. Difference was determined statistically significant if P<0.05.

## RESULTS

### Comparison of perioperative technical characteristics between the two groups

No difference was observed in the mode of valvular surgery, category of valve and length of hospital stay (P>0.05), but the duration of extracorporeal circulation and aortic occlusion of the observation group was significantly longer than that of the control group (P<0.05; Table-I).

**Table-I:** Comparison of perioperative technical characteristics between the two groups.

Item	Observation group	Control group	t /X2	P
Single-valve surgery (n)	44	37	0.459	>0.05
Double-valve surgery (n)	21	13		
Bioprosthetic valve (n)	9	7	0.052	>0.05
Mechanical valve (n)	36	26		
Duration of extracorporeal circulation (min)	126.41±12.34	112.63±8.38	17.664	<0.05
Duration of aorta occlusion (min)	93.32±7.84	87.14±6.85	9.387	<0.05
Postoperative length of hospital stay (d)	9.0±1.6	8.9±1.9	1.167	>0.05

### Comparison of curative effect between the two groups

The postoperative one-year follow-up suggested that the atrial fibrillation cardioversion rate of the observation group was 90.8% (59/65), (57/65) 87.7% and (55/65) 84.6% at postoperative month three, month six and year one respectively; the corresponding rate of the control group was 22.0% (11/50), 18.0% (9/50) and 14.0% (7/50) respectively. The difference of the cardioversion rate between the two groups at different time points had statistical significance (Table-II).

**Table-II:** Comparison of follow-up results between the two groups.

Group	Postoperative month 3			Postoperative month 6			Postoperative year 1		

	Atrial fibrillation cardioversion rate (%)	LVEF(%)	Inner diameter of the left atrium (mm)	Atrial fibrillation cardioversion rate (%)	LVEF(%)	Atrial fibrillation cardioversion rate (%)	Atrial fibrillation cardioversion rate (%)	LVEF(%)	Atrial fibrillation cardioversion rate (%)
Observation group	90.8*	61.6±5.5	47.3±5.4*	87.7*	62.4±5.1	43.8±5.1*	84.6*	62.5±5.1	38.7±4.7*
Control group	22.0	61.4±5.6	53.8±5.7	18.0	62.1±5.0	53.4±5.3	14.0	61.8±4.9	53.2±5.1

* ***Note:*** indicated that P<0.05 compared to the control group.

### Comparison of surgery associated adverse events between the two groups

All the patients in the observation group completed cardiac valvular surgery and atrial cryoablation without withdrawal, and all the patients in the control group completed cardiac valvular surgery. No patient in the two groups died, underwent re-thoracotomy, or had nervous system associated adverse events such as cerebral stroke and transient ischemic attack in the perioperative period. In the observation group, there was one case of low cardiac output syndrome (LCOS) and two cases of acute renal insufficiency; in the control group, there were one case of LCOS and one case of pulmonary infection. The difference of the postoperative complications between the two groups had no statistical significance (P>0.05).

## DISCUSSION

Atrial fibrillation is a common arrhythmia in clinics which can induce cardiac function decline and increase risks of cerebral stroke. The incidence of atrial fibrillation has been increasingly higher with the population aging. A latest study of Chef et al. demonstrated that the incidence of atrial fibrillation among Chinese people over 65 years was about 3.5%.^11^ In 2014, the Guidance of Treatment and Diagnosis of Atrial Fibrillation jointly released by American Heart Association, American College of Cardiology and Heart Rhythm Society definitely pointed that all patients with heart disease who underwent cardiac surgery in combination with persistent atrial fibrillation should undergo ablation treatment.^12^ Therefore adopting measures for timely control of atrial fibrillation has gradually been an important topic.

With the improvement of energy used in atrial fibrillation ablation in recent years, cryoablation has been gradually used in cardiac valvular surgery in combination with atrial fibrillation treatment as a new technology. The evaporation of refrigerant (nitrous oxide in liquid state) brings away a large amount of heat from tissues, which rapidly cools tissues and induces cell denaturation to damage cardiac muscle tissue or bypass which disturbs normal cardiacelectricalactivity and restore normal conduction of electrocardiogram.^13^-^15^ Ad N et al. did a follow-up study on 124 patients who underwent heart surgery and cryoablation in the same period and found that the death rate in perioperative period was lower than 2%,^16^ the incidence of cerebrovascular disease in perioperative period was lower than 1%, and the maintenance rate of sinus rhythm at postoperative year one was 87%. Yanagawa B et al. observed the effect of heart disease in combination with cryoablation of 250 patients.^17^ The average duration of atrial fibrillation of all the patients was (35.7±54.2) months, and 86% of them was non-paroxysmal atrial fibrillation. The average postoperative follow-up time was (28.2±23.7) months; the maintenance rate of sinus rhythm was 92.4% two years after surgery, among which 82.8% of them were patients with sinus rhythm who stopped taking anti-arrhythmia agent. Funatsu et al. retrospectively analyzed the clinical data of 268 patients who underwent mitral valve surgery and cryoablation simultaneously and found that the maintenance rates of sinus rhythm three years and five years after surgery were 84.1% and 80.2% respectively.^18^ All the above research results demonstrated that cryoablation was safe and effective in treating atrial fibrillation.

The results of the present study suggested that the observation group had long duration of aortic occlusion compared to the control group, but the extracorporeal circulation associated complications did not increase, and the length of hospital stay was not obviously extended. The maintenance rate of sinus rhythm of the observation group was 84.6% one year after surgery, remarkably higher than that of the control group, which was close to the results of other studies.^19^-^21^ It indicated that the surgery was safe and effective and could achieve high maintenance rate of sinus rhythm in short-term follow up.

Cryoablation is not recommended for the posterior annular of mitral valve blindly though it has minimal damages to the coronary artery.^22^ The best schemes for ablation lines and time should be formulated according to the structure of the left atrium and the thickness of the atrial wall. For example, ablation time should be reduced if the left atrium extends and the atrial wall is thin; the optimal line should be selected for some female patients with thin left atrial wall and soft myocardial interstitium to reduce damages to atrial walls. Moreover the area around the opening of superior vena cava should be avoided when ablation is performed in the right atrium. The above operation is also one of the reasons why there was no occurrence of grade III atrioventricular block after surgery in this study.

## CONCLUSION

We took the lead in carrying out simultaneous valve surgery and cryoablation in the treatment of atrial fibrillation in China and obtained definite results. It should be pointed out that the perioperative complications did not increase, and the recurrence rate of atrial fibrillation in the short-term follow up was low. Moreover cryoablation was performed along with minimally invasive incision and reoperation, which breaks through the poor radiofrequency ablation effect of atrial fibrillation with large left atrium. The safety and effectiveness of valve surgery in combination with cryoablation has been verified initially in this study; hence the therapy is worth promotion. However, the long-term effect remains to be further verified through prospective, large-scale and randomized controlled clinical studies because of the small sample size and short follow-up time of this retrospective study.

### Authors’ Contribution

**WWC, JH, & XPC:** Study design, data collection and analysis.

**WWC, JH, HW, SC, GJZ & XPC:** Manuscript preparation, drafting and revising.

**XPC:** Review and final approval of manuscript.
